# Horizons in Endometriosis: Proceedings of the Montreux Reproductive Summit, 14-15 July 2023

**DOI:** 10.52054/FVVO.16.s1.011

**Published:** 2024-04-11

**Authors:** A Vallée, E Saridogan, F Petraglia, J Keckstein, N Polyzos, C Wyns, L Gianaroli, B Tarlatzis, J.M. Ayoubi, A Feki, On behalf of the Montreux Reproductive Summit Working Group (alphabetic order)., B Ata, M Brännström, C Calhaz-Jorge, H.B. Carvalho Ferreira, R Campo, R.L. De Wilde, M Fehr Peter, A Fruscalzo, G Grimbizis, B Guani, T Gurgan, N Lambalk, I Lebbi, P Levi Setti, A Makrigiannakis, C Marti, G Moawad, T Motrenko Simic, M Mueller, M Nisolle, N Pluchino, C Racowsky, C Simon, E Somigliana, V Tanos, K Terras, C Tomassetti, D Wunder

**Affiliations:** Department of Epidemiology and Public Health, Foch Hospital, 92150 Suresnes, France; University College London, Elizabeth Garrett Anderson Institute for Women’s Health, London, United Kingdom. University College London Hospital, Women’s Health Division, London, United Kingdom; Department of Experimental, Clinical and Biomedical Sciences, University of Florence, Careggi University Hospital, Viale Morgagni 44, 50134, Florence, Italy; (SEF), Stiftung Endometrioseforschung, Westerstede, Germany. Gynecological Clinic, Gynecological Clinic Drs Keckstein, Villach, Austria. Department of Obstetrics and Gynecology, Ulm University Hospital, Ulm, Germany; Reproductive Medicine, Dexeus University Hospital, Barcelona, Spain. Faculty of Medicine and Health Sciences, Ghent University (UZ Gent), Gent, Belgium; Cliniques Universitaires Saint-Luc, Université Catholique de Louvain, Brussels, Belgium; SISMER, Reproductive Medicine Unit, Via Mazzini 12, 40138 Bologna, Italy; Professor Emeritus of Obstetrics – Gynecology and Human Reproduction, School of Medicine, Aristotle University of Thessaloniki, Greece; Department of Obstetrics, Gynaecology and Reproductive Medicine, Foch Hospital, Suresnes, France. Medical School, University of Versailles, Saint-Quentin-en-Yvelines (UVSQ), Versailles, France; Department of Obstetrics and Gynecology, HFR—Fribourg, Chemin des Pensionnats 2-6, 1708 Fribourg, Switzerland. Faculty of Science and Medicine, University of Fribourg, Fribourg, Switzerland; ART Fertility Clinics, Dubai, United Arab Emirates. Department of Obstetrics and Gynecology, Koc University School of Medicine, Istanbul, Turkey; Department of Obstetrics and Gynecology, Sahlgrenska University Hospital, SE-41345 Gothenburg, Sweden; Faculdade de Medicina da Universidade de Lisboa, Lisbon, Portugal; Gynecology Department, Centro Hospitalar e Universitário de Santo António, 4099-001 Porto, Portugal; Life Expert Centre, Schipvaartstraat 4, 3000 Leuven, Belgium; University Hospital for Gynecology, Pius Hospital, University Medicine Oldenburg, Carl von Ossietzky University, Germany; Frauenklinik, Kantonsspital Graubünden, Chur, Switzerland; 1st Department of Obstetrics & Gynecology, “Papageorgiou” Hospital, Aristotle University of Thessaloniki, 56403 Thessaloniki, Greece; Bahcesehir University, Faculty of Medicine, Department of Obstetrics and Gynecology Istanbul, Turkey; Obstetrics and Gynaecology, Amsterdam University Medical Center, Amsterdam, The Netherlands; Ob-Gyn and Fertility Private Clinic, Dream Center, Tunis, Tunisia; Department of Gynecology, Division of Gynecology and Reproductive Medicine, Fertility Center, IRCCS Humanitas Research Hospital, Rozzano, Milan, Italy, Department of Biomedical Sciences, Humanitas University, Pieve Emanuele, Milan, Italy; Department of Obstetrics and Gynecology, University of Crete Medical School, Heraklion, Greece; Department of Obstetrics and Gynecology, George Washington University, Washington, DC 20037, USA. The Center for Endometriosis and Advanced Pelvic Surgery, Washington, DC 22101, USA; Human Reproduction Center Budva, Budva, Montenegro; Department of Obstetrics and Gynaecology, University Hospital of Berne and University of Berne, 3010 Berne, Switzerland; Department of Obstetrics and Gynecology, Hospital the Citadelle, University of Liege, 4000 Liege, Belgium; Division of Gynaecology, Lausanne University Hospitals and the Faculty of Medicine of Lausanne, Lausanne, Switzerland; Department of Obstetrics, Gynecology, and Reproductive Medicine Hospital Foch Suresnes, France; Carlos Simon Foundation, INCLIVA Health Research Institute, Valencia, Spain; Infertility Unit, Fondazione IRCCS Ca’ Granda, Ospedale Maggiore Policlinico, Milan, Italy; Aretaeio Hospital and St Georges Medical School, Nicosia University, Nicosia, Cyprus; IVF Center of the Hannibal International Clinic, Tunis, Tunisia; Department of Obstetrics and Gynaecology, Leuven University Fertility Center, University Hospitals Leuven, Leuven, Belgium

**Keywords:** Endometriosis, prevention, biomarkers, classification, fertility, menopause, artificial intelligence, diagnosis, treatment

## Abstract

Endometriosis is a complex and chronic gynaecological disorder that affects millions of women worldwide, leading to significant morbidity and impacting reproductive health. This condition affects up to 10% of women of reproductive age and is characterised by the presence of endometrial-like tissue outside the uterus, potentially leading to symptoms such as chronic pelvic pain, dysmenorrhoea, dyspareunia, and infertility. The Montreux summit brought a number of experts in this field together to provide a platform for discussion and exchange of ideas. These proceedings summarise the six main topics that were discussed at this summit to shed light on future directions of endometriosis classification, diagnosis, and therapeutical management. The first question addressed the possibility of preventing endometriosis in the future by identifying risk factors, genetic predispositions, and further understanding of the pathophysiology of the condition to develop targeted interventions. The clinical presentation of endometriosis is varied, and the correlation between symptoms severity and disease extent is unclear. While there is currently no universally accepted optimal classification system for endometriosis, several attempts striving towards its optimisation - each with its own advantages and limitations - were discussed. The ideal classification should be able to reconcile disease status based on the various diagnostic tools, and prognosis to guide proper patient tailored management. Regarding diagnosis, we focused on future tools and critically discussed emerging approaches aimed at reducing diagnostic delay. Preserving fertility in endometriosis patients was another debatable aspect of management that was reviewed. Moreover, besides current treatment modalities, potential novel medical therapies that can target underlying mechanisms, provide effective symptom relief, and minimise side effects in endometriotic patients were considered, including hormonal therapies, immunomodulation, and regenerative medicine. Finally, the question of hormonal substitution therapy after radical treatment for endometriosis was debated, weighing the benefits of hormone replacement.

## Introduction

Endometriosis, a complex and chronic gynaecological disorder, affects millions of women worldwide, causing significant morbidity and impacting their reproductive health (Becker et al., 2022). As we gathered for the Montreux Reproductive Summit, we had a unique opportunity to explore the horizons of endometriosis and discuss key areas of interest and future advancements in its management.

Endometriosis afflicts women of reproductive age, with estimates suggesting that it affects up to 10% of women in this population (Missmer et al., 2004). The condition is characterised by the presence of endometrial-like tissue outside the uterus, commonly found on the pelvic organs and structures. Endometriosis is associated with a range of symptoms, including chronic pelvic pain, dysmenorrhea, dyspareunia, and infertility. These symptoms significantly impact the quality of life of affected women and pose a significant burden on healthcare systems (Smolarz et al., 2021). The annual costs of treating endometriosis are substantial and comparable to other chronic diseases such as diabetes (Armour et al., 2019; Gao et al., 2006; Soliman et al., 2018).

The Montreux Reproductive Summit provided a platform for experts and researchers to come together and exchange knowledge, experiences, and ideas on various aspects of endometriosis. This paper will present six topics that were explored in depth during the summit, shedding light on the future directions in endometriosis management. First and foremost, we posed the question: Is it possible to prevent endometriosis in the future? Endometriosis has long been associated with enigmatic origins and elusive risk factors (Vallée et al., 2024; Wang et al., 2020). However, recent advancements in our understanding of the disease have sparked interest in potential preventive strategies. By identifying risk factors, uncovering genetic predispositions, and further elucidating the pathophysiology of endometriosis, we may be able to develop targeted interventions to prevent the onset or progression of this condition.

The absence of a universally accepted staging system poses a significant challenge in the management of endometriosis. Over the years, several attempts have been made to improve endometriosis classification, but each system has its advantages and limitations. Hence, discussing the relevance of and developing a new endometriosis classification system on the light of existing classifications could eventually effectively facilitate communication among healthcare professionals and lead to standardised treatment approaches.

The clinical presentation of endometriosis is varied, and the correlation between symptom severity and disease extent is unclear. Turning our attention to proper diagnosis, we must contemplate the future of endometriosis diagnostic tools. While laparoscopy remains the gold standard for definitive diagnosis, it is an invasive and costly procedure (Hsu et al., 2010). Development of non-invasive and easily accessible diagnostic tools holds immense potential for improving patient care and reduce the diagnostic delay that often plagues endometriosis patients. By discussing emerging diagnostic techniques, such as biomarker analysis, ultrasound imaging, and artificial intelligence algorithms, we can envisage a future where early detection and timely management of endometriosis are within reach.

The burden of infertility is often a significant concern for women with endometriosis. Importantly, as both the disease and its treatment affect the ovarian reserve and may jeopardize the future reproductive potential, another crucial aspect of managing endometriosis patients is preserving their fertility (Llarena et al., 2019). Determining when and how to preserve fertility in these patients is thus essential to identify the best approaches for optimising future fertility outcomes in the context of endometriosis. By exploring the latest research and clinical strategies on fertility preservation, we can empower patients faced with endometriosis to make informed decisions about their reproductive futures.

Evolution in the therapeutic arsenal is awaited making it essential to share knowledge and experiences among experts present at the Montreux Reproductive Summit and focus on novel medical therapies for endometriosis Montreux Reproductive Summit. While hormonal and surgical interventions are currently available, they may not always be effective or suitable for all patients. Therefore, it is crucial to explore future medical therapies for endometriosis that can target the underlying mechanisms of the disease, provide effective symptom relief, and minimise side effects (Nothnick et al., 2018). By investigating potential new treatment modalities, such as targeted therapies, immunomodulation, and regenerative medicine approaches, we can envisage a future where endometriosis management is tailored to individual patients’ needs.

Lastly, the question of hormonal substitution therapy after radical treatment for endometriosis needed to be addressed (Al Kadri et al., 2009). The decision to use hormone replacement therapy (HRT) after surgical intervention for endometriosis poses a clinical challenge, as it involves weighing the benefits of hormone replacement against the potential recurrence or progression of the disease.

By exploring the available evidence and discussing individual patient factors, we can shed light on the best practices and considerations for hormonal substitution therapy in patients with endometriosis.

By collaborating and sharing our knowledge and experiences during this summit, we aimed to provide an overview of the current challenges in endometriosis care. This meeting gave us the opportunity to discuss how to shape the future of endometriosis care and improve the lives of millions of women worldwide.

## 1. Endometriosis: is it possible to prevent endometriosis in the future?

**Figure 1:**
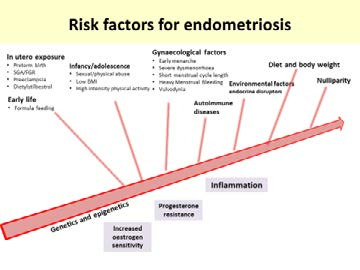
Lifelong risk factors for endometriosis (SGA: small for gestational age, FGR: fetal growth retardation).

There are a number of risk factors that have been associated with an increased future risk of endometriosis (Figure 1).

The genetic tendency predisposition for endometriosis has long been recognised. Endometriosis is 6-9 times more common in the presence of a first degree relative with endometriosis. It is thought that endometriosis may be a multigenetic hereditary condition with involvement of various pathways including hormones, inflammation, immunity, and pain. A recent genome-wide association study meta-analysis has identified 42 genome-wide significant loci comprising 49 distinct association signals for endometriosis. Genetic correlations are more important for advanced disease and ovarian endometriosis. The identified signals regulate expression or methylation of genes in endometrium and blood, many of which are related with pain perception (SRP14/BMF, GDAP1, MLLT10, BSN, NGF). A significant genetic correlation has also been observed between endometriosis and 11 pain-conditions including migraine, back, and multisite chronic pain (MCP), as well as inflammatory conditions including asthma and osteoarthritis (Rahmioglu et al., 2023).

Understanding the epigenetic regulation of gene expression in endometriosis provides valuable insights into the disease pathogenesis and progression. Alterations in DNA methyl transferase (DNMT) expression levels have been observed in endometriotic tissue and aberrant DNA methylation, and histone modifications contribute to progesterone resistance and dysregulation of gene expression in endometrial tissue and developing lesions (Psilopatis et al., 2023). Perinatal and early-life events such as preterm birth, preeclampsia, low birth weight and formula feeding have been suggested as potential risk factors for the future development of endometriosis (Vannuccini et al., 2016).

History of chronic pain including headache and migraine, vulvodynia and stress is more common in women with endometriosis. Chronic stimulation of peripheral nerves and central nervous system can lead to peripheral and central sensitisation and eventually neuropathic type pain. Headache and migraine are associated with endometriosis in adolescence, in particular menstrual-related migraine and headache symptoms start years before the diagnosis of endometriosis (Pasquini et al., 2023). The sensory fibres from ectopic endometrial implants can lead to neuronal hyperactivity, potentially triggering migraine attacks (Wu et al., 2022). A strong genetic overlap between endometriosis and migraine has been shown (Adewuyi et al., 2020; Rahmioglu et al., 2023).

Later in life, early menarche, dysmenorrhoea, heavy menstrual bleeding, and presence of genital tract abnormalities are identified as risk factors. Primary dysmenorrhoea is often associated with endometriosis (Hewitt, 2020) and endometriosis itself is a leading cause of dysmenorrhoea, particularly secondary dysmenorrhoea (Sachedin and Todd, 2020). Primary dysmenorrhoea (PD) affects a significant number of adolescents and young women and is often overlooked as a sign of endometriosis as these young women may consider the pain to be a normal part of their menstrual cycle and fail to report it or seek medical care (Clemenza et al., 2021). It has a significant impact on women’s lives, leading to restrictions in daily activities, absenteeism from school and sport, lower academic performance in adolescents, poor sleep quality, and negative effects on mood, including anxiety and depression. It also results in a loss of productivity for society (Clemenza et al., 2021). It is often treated with non-steroidal anti-inflammatory drugs or combined oral contraceptives (COCs), but according to an American College of Obstetricians and Gynecologists (ACOG) committee report (American College of Obstetricians and Gynecologists, 2018) when a patient does not experience clinical improvement for dysmenorrhoea within 3–6 months of therapy initiation possible secondary causes such as endometriosis must be investigated (Hewitt, 2020; Hirsch et al., 2020).

Genital tract anomalies were associated with endometriosis in adolescence which incidence lies between 11-40%; with the mostly reported Mullerian anomalies being those associated with outflow tract obstruction such as unicornuate uterus with rudimentary horn, or uterine didelphys with obstructed hemivagina (Borghese et al., 2015; Dovey and Sanfilippo, 2010). But early age at menarche and menstrual disorders such as heavy menstrual bleeding, short menstrual cycle length, and long menstrual flow (≥ 6 days) were associated with a higher risk of endometriosis (Giudice et al., 2022; Nnoaham et al., 2012).

A potential association between stress and the development and progression of endometriosis is suggested (Reis et al., 2020a). Women with endometriosis experience increased stress levels, psychological and endocrine stress measures indicating that there is a correlation with pain severity and disease extension. Nevertheless, chronic stress might be a primary cause of endometriosis (at least in animal model), and, consequently, avoiding or treating chronic stress might potentially reduce the risk of developing endometriosis (Reis et al., 2020a)

Psychological stress may be found in the history of women with endometriosis. Childhood stress, including neglect and abuse, may contribute to the development of endometriosis (Fuentes and Christianson, 2018). Abuse severity, chronicity, and accumulation of types of abuse are associated with increasing risk of endometriosis in a dose– response manner. Furthermore, this association is stronger among women who never report infertility and at the same time are symptomatic with respect to pain (Harris et al., 2018). History of sexual abuse during childhood and/or adolescence is associated with the presence of severe pelvic pain symptoms irrespective of the presence of endometriosis (Bourdon et al., 2023).

An association among low body mass index, strenuous physical activity, and endometriosis has also been reported (Lafay Pillet et al., 2012; Vitonis et al., 2009).

An increased risk of endometriosis has been also shown in women with autoimmune diseases including systemic lupus erythematosus, Sjögren’s syndrome, rheumatoid arthritis, autoimmune thyroid disorder, coeliac disease, multiple sclerosis, inflammatory bowel disease, and Addison’s disease, although more studies are necessary because there are few high-quality studies (Kvaskoff et al., 2015; Shigesi et al., 2019).

Environment, food, and consumer products may influence the development of endometriosis; they may interfere with hormone biosynthesis, metabolism, or action, thereby influencing reproduction and endometriosis (Cobellis et al., 2003; Markowska et al., 2023). Polyhalogenated aromatic hydrocarbons (PHAH), a class of widespread environmental contaminants are linked to endometriosis. Dioxin (2,3,7,8-tetrachlorodiben- zo-p-dioxin) or Di-(2-ethylhexyl)-phthalate (DEHP), commonly used plasticiser in ¯flexible polyvinylchloride (PVC) formulations are found in plasma and in the peritoneal fluid of women with endometriosis (Cobellis et al., 2003). Diet can significantly impact on the progression of endometriosis by oestrogen action or inflammatory processes. Polyphenols are an extensive group of biologically active compounds synthesised by plants and there is structural similarity between these compounds and oestradiol or the synthetic oestrogen diethylstilbestrol (Markowska et al., 2023).

One of the most important risk factors for endometriosis is the postponed first pregnancy. In fact, pregnancy improves symptoms and endometriosis lesions may undergo decidualisation or regression. In the course of the last century delayed first pregnancy and reduced has resulted in a lack of a protective factor for endometriosis (Moen and Muus, 1991).

In the presence of identifiable risk factors including dysmenorrhoea and/or chronic pelvic pain and with additional use of imaging and potential biomarkers, it may be possible to implement management strategies to prevent development and/ or progression of endometriosis. These strategies may involve using hormonal contraceptives, progestins and alternative approaches such as a healthy diet and lifestyle, relaxation techniques, physiotherapy, cognitive behavioural therapy, acupuncture, osteopathy, and nerve stimulation techniques. Future studies are needed to explore whether these approaches are effective.

## 2. Is there a need for new endometriosis classification?

The search for a universally accepted staging system poses a significant challenge in the management of endometriosis. A reliable and well- structured classification system is essential not only for facilitating effective communication among healthcare professionals but also for establishing standardised treatment approaches. Currently, the effectiveness of existing classification systems remains a subject of controversy. The clinical presentation of endometriosis is varied, and the correlation between symptom severity and disease extent is unclear. Over the years, several attempts have been made to improve the classification of endometriosis.

In this section, an overview of some of the most frequently used classification systems for endometriosis is given.

### 2.1. The American Society for Reproductive Medicine classification

#### 2.1.1. Definition

In 1979, the American Fertility Society (AFS) introduced the AFS score, a scoring system to assess the stage of endometriosis (American Fertility Society, 1979). This system assigned weighted values based on the size of endometriotic lesions in the ovaries, peritoneum, and fallopian tubes, as well as the severity of adhesions at these sites. The stages were categorised as I (mild), II (moderate), III (severe), and IV (extensive). However, critics pointed out that this classification system lacked a correlation between disease stage and clinical symptoms such as pain and infertility (Hasson, 1981; Lee et al., 2020).

The revised system, renamed the revised American Society for Reproductive Medicine (rASRM) classification in 1996, gained global acceptance (American Society for Reproductive Medicine, 1997).

#### 2.1.2. Limitations

The rASRM classification has some drawbacks. rASRM only considers endometriosis sites in the pelvis, especially those that can be visualised by diagnostic laparoscopy (peritoneum, ovary, tube, uterosacral ligament (USL) and adhesions). Deep endometriosis (DE), especially extragenital and extraperitoneal DE, is not considered. This could potentially explain the lack of correlation between symptoms and stage. In addition, classification of surgical findings into four stages using this ‘not quite simple system’ can only be applied in a useful and correct way with an electronic programme (Metzemaekers et al., 2020).

Furthermore, there is discrepancy between histologically diagnosed endometriosis and visually diagnosed stage. A study comparing the pathologic findings of surgically removed endometriosis with visually diagnosed rASRM stages found that concordance rates were lower for stage I disease, indicating a higher likelihood of misdiagnosis based on visual inspection (Fernando et al., 2013). Secondly, the reproducibility of the rASRM score is poor among different observers and even within the same observer. The severity of pain and infertility does not consistently correlate with the rASRM stage. Pain symptoms and deep dyspareunia were not consistently related to the stage of endometriosis, and the presence of vaginal lesions was more frequently associated with severe deep dyspareunia (Hornstein et al., 1993). Pregnancy rates did not show significant differences among different stages, except for a slight decrease in stage IV endometriosis (Guzick et al., 1982).

### 2.2. The ENZIAN classification

#### 2.2.1. Definition

**Figure 2:**
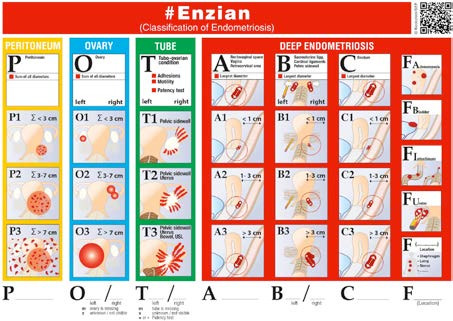
The #ENZIAN classification.

The ENZIAN classification was introduced in Germany, Austria, and Switzerland in 2005, initially as a supplementary system to the rASRM classification, focusing on DE (Tuttlies et al., 2005). The ENZIAN classification divides retroperitoneal structures into three compartments (A, B, and C) and grades the severity of the lesion based on invasiveness, and includes extragenital lesions (bowel, bladder, ureter and extrapelvic localisations). The latest version of the classification, now called the #Enzian classification, takes into consideration all endometriosis lesions, and makes an additional use of the rASRM unnecessary (Keckstein et al., 2021). The #ENZIAN classification includes a detailed description of all anatomical structures (Figure 2). This classification can, with some limitations, provide a much more comprehensive description of the disease location and extent, thus providing a clearer assessment of the extent and severity of endometriosis than is possible with the rASRM (Montanari et al., 2022). The great advantage of the classification lies in its applicability for both non-invasive and invasive diagnostics. The high accuracy of sonographically performed classification with surgical findings has been demonstrated in several studies. #Enzian can also be used in the context of magnetic resonance imaging (MRI) reporting (Maciel et al., 2023; Harth et al., 2023). Enzian can predict the extent of the disease with high accuracy before surgery (Di Paola et al., 2015) and has shown associations with the presence and severity of symptoms, particularly in relation to pain and surgical complexity (Johnson et al., 2017; Thomassin-Naggara et al., 2020).

#### 2.2.2. Limitations

The ENZIAN classification also faces limitations. Sufficient knowledge of pelvic anatomy, both in diagnostic and surgical diagnostics, requires advanced expertise from the clinician. In addition, if surgery is incomplete, it may be necessary to combine the results of the different diagnostic procedures (i.e. ultrasound and/or MRI). The utility of the preoperative #ENZIAN score in evaluating prediction of fertility requires further investigation.

### 2.3. The Endometriosis Fertility Index

#### 2.3.1. Definition

The EFI (Endometriosis Fertility Index) system was developed to predict pregnancy rates in infertile patients with surgically diagnosed endometriosis who have not undergone in vitro fertilisation (IVF) (Adamson and Pasta, 2010). The EFI system incorporates both historical factors (such as age, duration of infertility, and previous pregnancies) and surgical factors (including scores describing the condition of the fallopian tubes, their fimbriae, and the ovaries – defined as ‘least function score minus the rASRM total score). The least function score assesses the functionality of the fallopian tubes, fimbriae, and ovaries, while other surgical factors evaluate the severity of endometriosis lesions. The EFI system offers a clear advantage in predicting pregnancy outcomes compared to the rASRM classification.

#### 2.3.2. Limitations

The EFI does not correlate with pain symptoms, which are an important aspect of endometriosis. Additionally, the subjective judgment involved in assigning the least function score can introduce variability among surgeons, and the system is more complex than the rASRM classification and ENZIAN score, requiring calculations and score additions. Further studies are needed to evaluate the interobserver reliability and intraobserver reproducibility of the EFI system and to explore its utility in assessing IVF outcomes in endometriosis patients.

### 2.4. The American Association of Gynecologic Laparoscopists classification

#### 2.4.1. Definition

In 2007, the AAGL (American Association of Gynecologic Laparoscopists) initiated a project to develop a new classification system for endometriosis (Abrao et al., 2021). The system involved the participation of 30 endometriosis experts who assigned scores ranging from 0 to 10 based on the significance of each site of involvement in relation to pain, infertility, and surgical complexity. This comprehensive system aimed to quantify the extent of the disease and consider important factors for patient assessment. Surgical difficulties were categorized into four levels, with increasing complexity and involvement of specific organs. Validation of the classification system involved collecting visual analogue scale scores and infertility history from patients prior to surgery.

#### 2.4.2. Limitations

The AAGL classification has not been fully validated despite more than 10 years since its proposal. Further investigation and discussion are necessary to fully evaluate and establish the validity and usefulness of the AAGL classification in clinical practice.

### 2.5. Revitalising Endometriosis Classification: A Call for a New, Appropriate, and Validated System

To date, each classification system has its own advantages and limitations. The ideal classification should accurately describe the sites and extent of the disease, correlate closely with endometriosis symptoms (pain and infertility), reflect the surgical difficulty based on the location of the disease, and be user-friendly for surgeons, validated for both pain and infertility, and establish a comprehensive universal language for clinical practitioners and researchers to facilitate collaboration and enhance understanding of the disease (Lee et al., 2020).

A working group of the European Society for Gynaecological Endoscopy (ESGE), The European Society of Human Reproduction and Embryology (ESHRE) and World Endometriosis Society (WES) published recommendations for the surgical treatment of DE, highlighting the importance of classifying DE lesions (Working group of ESGE, ESHRE, and WES et al., 2020) and recommended documenting specific information about the location, size, and involvement of adjacent organs and structures.

While no gold standard for endometriosis classification currently exists, expert consensus has been reached on the need to utilise the available systems. The World Endometriosis Society (WES) released a consensus statement in 2017, suggesting that the rASRM classification should be completed by all women undergoing surgery to obtain maximum information. For women with DE, the ENZIAN classification should additionally be completed, and the EFI system should be considered for women who need to consider fertility in the future. These recommendations provide guidance until a better classification system becomes available (Johnson et al., 2017). The #Enzian classification comes much closer to this comprehensive idea. It is therefore recommended in a consensus paper by eight international gynaecological and imaging societies for use in non-invasive diagnostics. However, there are still several aspects to be assessed (Condous et al., 2024).

The significant improvement of non-invasive diagnostics, surgical therapy and multimodal interdisciplinary therapy concepts require a uniform common language with high accuracy and information. This will allow much more precise scientific research into this very unclear disease. In the future, computer systems combined with artificial intelligence (AI) will also significantly reduce the problem of the complexity of the various classification systems and improve the benefit for their clinical and scientific application.

Additional information about symptoms, histological findings and clinical course data etc. could then be usefully integrated if necessary.

## 3. Which future endometriosis diagnostic tools for tomorrow?

### 3.1. The clinical problem

The diagnosis of endometriosis is often delayed, with an average diagnostic delay of up to 12 years (Aubry et al., 2023). This delay is primarily due to several factors. Firstly, the symptoms of endometriosis are often not readily recognised in primary care settings, leading to misdiagnosis or dismissal of symptoms (Hudson, 2021). Secondly, women may normalise their symptoms over time, attributing them to normal menstrual discomfort (Sachedin and Todd, 2020). This normalisation further contributes to the delay in seeking medical help.

Another factor contributing to the diagnostic delay is the premature exclusion of endometriosis based on negative transvaginal ultrasound results (Nisenblat et al., 2016a). Endometriosis lesions can be challenging to detect using ultrasound alone, especially in cases where the lesions are small or located in deeper pelvic areas (Hsu et al., 2010). As a result, negative ultrasound findings may lead to the dismissal of endometriosis as a possible cause of symptoms (Pascoal et al., 2022). In addition, potential risks of a surgical intervention to confirm or rule out diagnosis may be a discouraging factor amongst clinicians and patients.

Furthermore, some women may experience temporary relief of symptoms using oral contraceptives or during pregnancy. This symptom cessation can further delay the recognition and diagnosis of endometriosis, as the temporary relief may lead healthcare providers to overlook the possibility of the disease (Weisberg and Fraser, 2015).

The diagnostic delay in endometriosis is concerning due to its significant impact on patients. When patients are finally diagnosed, more than 90% of them have moderate to severe symptoms (Bazot et al., 2017). The disease can progress over many years, leading to greater treatment costs, prolonged negative impact on quality of life, including central sensitisation, psychological well- being, and increased risks of surgical interventions and infertility. This emphasises the urgent need for a non-invasive diagnostic test that can aid in the earlier detection of endometriosis.

Developing novel and non-invasive methods improving existing approaches to reliably detect or exclude endometriosis is of paramount importance. Such advancements would help reduce the diagnostic delay, allowing for earlier interventions and improved management of the disease. It would lead to better outcomes for patients, including timely access to appropriate treatments and support, and potentially prevent the progression of endometriosis-related complications.

### 3.2. Diagnostic test accuracy

Diagnosing endometriosis involves assessing both its presence or absence and the extent of the disease (Nisenblat et al., 2016a). Laparoscopy is considered the gold standard for diagnosing endometriosis by several groups and guidelines (Johnson et al., 2013). It allows for direct visualisation of endometriosis lesions and histological assessment through biopsy. However, the requirement for surgical and histological diagnosis has led to delays in accessing treatment. To address this, there has been a shift towards prescribing empirical medical therapy before or instead of laparoscopy, except when fertility is a priority (Rolla, 2019). This approach, known as clinical diagnosis, combines the clinical history and physical examination. However, clinical diagnosis is controversial due to its poor diagnostic performance and the resulting uncertainty among patients and healthcare providers (Abrao et al., 2007; Wykes et al., 2004).

Imaging techniques such as transvaginal ultrasound (TVS), transrectal ultrasound (TRS), and MRI can bridge the gap between clinical and surgical diagnosis (Alborzi et al., 2018). These non-invasive methods provide a visual diagnosis that is quicker, safer, and more accessible than surgery. However, imaging-based diagnosis also has its challenges and controversies.

Diagnosing endometriosis involves more than determining its presence or absence; considering the subtype, location, and extent of the disease is also crucial for clinical management (Pascoal et al., 2022). Endometriosis can manifest in non- gynaecological organs, further complicating diagnosis and treatment planning. Understanding the extent of the disease before surgery is important to avoid incomplete or suboptimal resection, which can lead to persistent pain and complications.

When assessing the accuracy of diagnostic tests for endometriosis, sensitivity and specificity are important metrics. However, their interpretation can be challenging. The positive predictive value (PPV) and negative predictive value (NPV) of a diagnostic test depend on disease prevalence. The likelihood ratio (LR) is a useful measure to assess the utility of a diagnostic test, as it considers the likelihood of a given test result in patients with or without the disease (Pascoal et al., 2022).

Comparing novel diagnostic modalities to the current gold standard methods is essential. Direct visualisation and histopathology have traditionally been considered the gold standard for endometriosis diagnosis. Skill levels of healthcare providers performing the tests should be considered, as test accuracy relies on their expertise. Verification bias is another challenge in endometriosis diagnostic research, as not all patients undergo laparoscopy.

Clinical diagnosis of endometriosis is based on signs, symptoms, and physical examination. It involves taking a detailed clinical history and performing a pelvic examination. This shift in diagnosis focuses on the patient rather than solely on identifying lesions during surgery. Clinical diagnosis has been shown to decrease diagnostic delay and allows for earlier confirmation and initiation of treatment.

In summary, diagnosing endometriosis involves considering its presence or absence, as well as the extent of the disease. Laparoscopy is currently the gold standard, but clinical diagnosis and imaging techniques like TVS, TRS, and MRI offer non-invasive alternatives. Understanding the accuracy of diagnostic tests and their role in clinical decision-making is crucial for effective management of endometriosis.

**Table I:** Summary of strengths, limitations and reported diagnostic accuracy of different endometriosis diagnostic methods.

Diagnostic modality	Strengths	Limitations	Diagnostic accuracy
Clinical history	Non-invasive Feasible, low-cost Symptomatology can predict disease location May facilitate therapeutic adherence May guide treatment choice, depending on complaints	Common symptoms of endometriosis have wide differential diagnosis Symptoms not predictive of disease extent	Se, 76–98% (Eskenazi et al., 2001; Nawrocka-Rutkowska et al., 2021); Sp, 20–58% (Eskenazi et al., 2001; Nawrocka-Rutkowska et al., 2021)
Physical examination	Accessible High specificity Opportunity to detect DE by visualisation or palpation	Low sensitivity Outcomes are operator-dependent Diagnostic accuracy varies by disease location Examination may be considered invasive and painful	Se, 18–88% (Bazot et al., 2009; Eskenazi et al., 2001; Hudelist et al., 2011) Sp, 76–100% (Eskenazi et al., 2001; Hudelist et al., 2011)
Biomarkers	Objective measure Combination may rule in endometriosis as a triage test (further research required)	Dependent on laboratory techniques and quality control protocols Some vary with hormonal and menstrual fluctuations Some are not specific to endometriosis Cannot discern DE, OE or SE	Anti-endometrial antibodies: Se, 81%; Sp, 75% (Nisenblat et al., 2016b) IL-6: Se, 63%; Sp, 69% (Nisenblat et al., 2016b) CA 19-9: Se, 36%; Sp, 87% (Nisenblat et al., 2016b) CA 125: varies by cut-off used (Nisenblat et al., 2016b)
Ultrasound	High specificity and sensitivity for OE Overall high accuracy in detecting DE and POD obliteration Dynamic nature for organ mobility Allows anatomic mapping Opportunity to provide visual evidence to patients High tolerability Cost-effective	Limited ability to detect SE Detection of DE requires highly trained sonographers/sonologists Outcomes are operator-dependent Examination may be considered invasive and painful	SE: Se, 65–79%; Sp, 91–95% (Nisenblat et al., 2016a) OE: Se, 93%; Sp, 96% (Nisenblat et al., 2016a) DE: Se, 79%; Sp, 94% (Nisenblat et al., 2016a)
MRI	Images obtained appear the same to all viewers Overall high accuracy in detecting DE and extrapelvic endometriosis Allows anatomic mapping Opportunity to provide visual evidence to patients	Static assessment Limited ability to detect SE Variable imaging protocols reported in literature Low accuracy in defining OE: Se, 95%; Sp, 91% (Nisenblat et bowel depth of invasion Requires specific training endometriosis No consensus on how to describe findings High cost compared with ultrasound	SE: Se, 79%; Sp, 72% (Nisenblat et al., 2016a) OE: Se, 95%; Sp, 91% (Nisenblat et al., 2016a) DE: Se, 94%; Sp, 77% (Nisenblat et al., 2016a)
Laparoscopy	Overall high accuracy, considered gold standard Allows concomitant diagnosis and treatment Opportunity to provide visual evidence to patients Significant placebo effect	Invasive, carries surgical risk Diagnostic accuracy dependent on surgical experience Visual diagnosis challenged by heterogeneous lesion appearance, inaccessible lesions	Se, 90–94% (Gratton et al., 2022; Wykes et al., 2004); Sp, 40–79% (Gratton et al., 2022; Wykes et al., 2004)
Histology	Ultimate confirmation of diagnosis Can rule out other conditions Can diagnose without visual confirmation	Obtaining tissue for histology requires surgical excision Influenced by surgical environment and method of resection	NA

DE, deep endometriosis; MRI, magnetic resonance imaging; OE, ovarian endometriosis; POD, pouch of Douglas; SE, superficial endometriosis; Se, sensitivity; Sp, specificity.

**Table II:** Other tests with accuracies.

Biomarkers	Se	Sp	References
Antiendometrial antibodies	81%	75%	(Pascoal et al., 2022)
IL-6	63%	69%	(Pascoal et al., 2022)
CA 19-9	36%	87%	(Pascoal et al., 2022)
CA 125	No cut-off	No cut-off	

Se, sensitivity; Sp, specificity.

### 3.3. New biomarkers

#### 3.3.1 Biomarkers for fundamental mechanisms of endometriosis

The changes in the number and function of immunological components in endometriotic patients lead to an increase in the volume of peritoneal fluid (PF) (Heidari et al., 2021). The main cells present in PF are mononuclear cells, particularly macrophages, which make up about 85% of the cells (Králíčková and Vetvicka, 2015). These cells are more likely to cause inflammation and contribute to the development of endometriosis rather than control it. Additionally, endometriosis affects the expression of genes and proteins in both eutopic and ectopic endometrial stromal cells (EuESCs and EESCs, respectively) (Delbandi et al., 2013). Mononuclear cells, EuESCs, and EESCs release cytokines and growth factors that can affect themselves and other cells, including macrophages. These factors promote the proliferation, angiogenesis, and invasion of endometrial cells, which are fundamental mechanisms in the development of endometriosis (Lousse et al., 2012).

- One of the factors involved in these processes is monocyte chemoattractant protein-1 (MCP-1), a chemokine that activates and recruits’ macrophages and other mononuclear cells to secrete growth factors and cytokines (Ulukus et al., 2009). MCP-1 also stimulates the proliferation and maintenance of endometrial cells in ectopic sites, suggesting its involvement in the pathogenesis of endometriosis (Heidari et al., 2021).
- Hepatocyte growth factor (HGF) has been shown to affect monocytes and macrophages, leading to modulate inflammation. HGF has various effects on epithelial and endothelial cells, including proliferation, migration, extracellular matrix production, and tubulogenesis (Khan et al., 2008).
- Insulin-like growth factor-1 (IGF-1) is another mitogenic factor secreted by macrophages and other mononuclear cells. Recent studies have shown that EESCs can express the IGF-1 receptor (Gunter et al., 2008).

Several studies have reported increased concentrations of MCP-1, HGF, and IGF-1 in the peritoneal fluid and serum of endometriotic patients compared to controls (Ahn et al., 2015; Heidari et al., 2021). However, some studies have failed to show significant differences in the concentrations of these factors between women with and without endometriosis (Drosdzol- Cop et al., 2012; Vodolazkaia et al., 2012). The disease is complex and involves immune system defects both locally and systemically. Retrograde menstruation, which is the backward flow of menstrual blood into the peritoneal cavity, is a widely accepted theory for the development of intraperitoneal and ovarian endometriosis (Sourial et al., 2014). However, it does not explain either the less common locations of endometriosis such as remote areas, which may involve bone marrow- derived stem cells, or epigenetic factors that are involved. The microbiome may also play a role in the pathogenesis of endometriosis (D’Alterio et al., 2021).

Macrophages play a role in tissue remodelling during endometriosis development (Capobianco and Rovere-Querini, 2013). Studies have shown a progressive decrease in M1 macrophages and a progressive increase in M2 macrophages from the early to advanced stages of endometriosis (Laganà et al., 2020). Invariant natural killer T cells (iNKT) may also be involved in the pathogenesis of endometriosis (Correa et al., 2022).

Chemokines and immune receptors play a crucial role in the development and progression of endometriosis by promoting proliferation, angiogenesis, invasion, and decreased apoptosis of ectopic cells (Nishida et al., 2011).

In the context of biomarkers, perhaps we could consider the studies of Bendifallah et al on salivary microRNA signature for diagnosing endometriosis (Bendifallah et al., 2023).

#### 3.3.2. Metabolomics of the follicular fluid in endometriosis

The human follicular fluid plays a crucial role in follicle development, oocyte maturation, and IVF outcomes (Da Broi et al., 2018). It is composed of proteins, steroid hormones, lipids, and other metabolites. The components of follicular fluid can originate from various sources such as granulosa cells, theca cells, oocytes, and blood plasma transferred through the thecal capillaries.

Metabolomics, or metabolomic profiling, is a technique that quantitatively measures a large number of low molecular weight molecules in a sample, including bodily fluids, tissues, and breath exhalate (Dabaja et al., 2022). It is a powerful tool used to predict and measure biochemical activities within cells and has been proven useful in disease screening, diagnosis, characterisation, and monitoring. Metabolomic biomarkers can be single molecules or patterns of molecules that anticipate a clinically relevant endpoint. Metabolomic profiling can provide valuable insights into follicular fluid composition and its association with oocyte quality and IVF outcomes.

Differences in metabolomic profiles of the follicular fluid of infertile women, including those suffering from endometriosis were reported (Dabaja et al., 2022). Phosphatidic acids (PAs) are involved in several pathophysiological processes and are overproduced in endometriosis, potentially affecting pregnancy outcomes (Li et al., 2018). They may serve as biomarkers for infertility associated with endometriosis. Similarly, uterine factors such as myomas (fibroids), polyps, and adhesions can negatively impact pregnancy outcomes. Alterations in phospholipids such as phosphatidylethanolamine (PE) and phosphatidylglycerol (PG) are associated with uterine factor infertility, providing insights into the pathophysiological processes and potential therapeutic targets (Li et al., 2018).

Unexplained infertility refers to cases where no clinical diagnosis is found despite standard investigations. In these cases, the metabolic profile of women with unexplained infertility differs from control groups (Jayaraman et al., 2014). Abnormal levels of phosphatidylinositol (PI) and imbalance in PI3K (phosphatidylinositol 3-kinase) levels may negatively affect ovulation and nidation processes, contributing to infertility (Dabaja et al., 2022).

In summary, metabolomic analysis of follicular fluid and other biomarkers can provide valuable insights into the factors influencing oocyte quality, embryo selection, and pregnancy outcomes in IVF procedures. Understanding the metabolic profile and potential biomarkers associated with different infertility factors can help optimize IVF protocols and improve success rates.

#### 3.3.3. VOCs in follicular fluid

Volatilomics, a subgroup of metabolomics, focuses on volatile organic compounds (VOCs) that can be derived from both exogenous and endogenous sources (Brinca et al., 2022). VOCs are present in readily accessible biofluids such as urine, exhaled breath, saliva, blood, serum, skin emanations, breast milk, and tissues (Brinca et al., 2022). This approach provides insights into the physiological processes of various disorders, including cancer, genetic and metabolic disorders, schizophrenia, and infectious diseases (Longo et al., 2021). VOC signatures have been connected to these pathologies and hold the potential to serve as biomarkers (Spratlin et al., 2009).

In the context of endometriosis, volatilomics studies have revealed a versatile profile of specific compounds present in follicular fluid. These compounds, such as fatty aldehydes and siloxanes, show altered levels in women with endometriosis compared to controls (Brinca et al., 2022). The presence of these metabolites suggests possible disruptions in steroidogenesis and sphingolipid metabolism, as well as implications in cell signalling and apoptosis (Brinca et al., 2022).

The presence of the metabolite 4-methyl-2,4- bis(4-hydroxyphenyl)pent-1-ene is significantly higher in the follicular fluid of women with endometriosis compared to controls (Rehan et al., 2015). This compound is a phthalate metabolite that exhibits potent oestrogenic activity and may interfere with hormonal function and endocrine pathways (Hirao-Suzuki et al., 2021).

Other metabolomic studies using different analytical techniques, such as nuclear magnetic resonance (NMR) and gas chromatography- mass spectrometry (GC-MS), have also provided insights into the metabolic alterations associated with endometriosis and PCOS (Gongadashetti et al., 2021).

Overall, metabolomic including volatilomic approaches offer valuable tools for understanding the metabolic perturbations in diseases such as endometriosis. These techniques provide a comprehensive view of the metabolite profiles and potential biomarkers, contributing to the development of improved diagnostic and therapeutic strategies for this condition.

#### 3.3.4. Artificial Intelligence and endometriosis

In the past 5 years, the rapid emergence of artificial intelligence (AI) in healthcare has shown great potential in disease diagnostics, treatments, and analysis of biomedical datasets (Sivajohan et al., 2022; Wang et al., 2019). AI, particularly machine learning (ML), has been applied to various types of data, including multi-omics, clinical, behavioural/ wellness, environmental, and research and developmental data (Wang and Preininger, 2019). It has been used for decision-making, patient self-management, triage, understanding disease mechanisms, and drug discovery. However, AI methods require expert oversight to inform model development due to the complexity of clinical problems. Privacy and security of patient data also need to be considered when introducing AI technology into healthcare.

In obstetrics and gynaecology, AI technologies have been applied in areas such as foetal heart monitoring and reproductive medicine, showing potential in outcome prediction (Elgendi et al., 2020). Endometriosis, with its complex diagnostic challenges, can benefit from AI by improving non- invasive diagnostics and reducing delays and human error in diagnosis (Wang et al., 2010). However, clinicians face challenges in understanding different AI methods and the competencies and limitations of AI technologies.

With regards to endometriosis management, AI interventions can be used for various purposes, methodologies, and input types, including biomarkers, clinical variables, genetic variables, and metabolite spectra (Bouaziz et al., 2018; Lee et al., 2019; Matta et al., 2020).AI interventions have shown promising results in improving diagnostics, research efficacy, and outcome prediction in endometriosis (i.e. pooled sensitivity ranged between 81.7 and 96.7% and pooled specificity ranged between 70.7 and 91.6%.) (Sivajohan et al., 2022). However, the heterogeneity of study designs, input data, and AI interventions makes it challenging to compare accuracy and efficacy across different models.

Although AI technologies have the potential to reduce diagnostic errors and provide superior outcome prediction, many studies lack human comparators and fail to compare performance of AI with existing decision tools and clinical diagnostics. Standardised guidelines for ML applications in medicine are needed. Future studies should focus on comparing AI models with existing diagnostic methods and ensuring transparent descriptions of modelling methodology.

#### 3.3.5. Microbiome

Although many bacteria in the vagina are Lactobacilli, studies have found the presence of other bacteria, including Fusobacterium nucleatum, which may contribute to vaginal dysbiosis (Payne et al., 2021). Fusobacterium species are commonly found in the oral and gastrointestinal microbiota and have a symbiotic relationship with their hosts (Brennan and Garrett, 2019). While the uterine cavity is typically considered almost sterile, there is a known association between endometriosis and microbial colonisation (Khan et al., 2014). Recent investigations have shown that although the number of bacteria in the uterus is much lower than in the vagina, certain microbial communities, including Fusobacterium, can be detected (Chen et al., 2017; Muraoka et al., 2023). The exact reason why Fusobacterium selectively infects the endometria of some patients remains unclear, but there is evidence suggesting haematogenous transmission during pregnancy or transmission through the vagina (Vander Haar et al., 2018). Notably, the presence of Fusobacterium in vaginal swab samples from patients with endometriosis was significantly higher compared to those without endometriosis, supporting the possibility of a vaginal transmission route (Muraoka et al., 2023). Fusobacterium, such as F. nucleatum, has been found to damage the intestinal barrier and induce aberrant inflammation (Liu et al., 2019). These pathogenic roles may be attributed to Fusobacterium’s strong adhesion to epithelial tissues and its invasive abilities (Strauss et al., 2011).

In vitro experiments demonstrated that even heat-killed F. nucleatum effectively stimulated the production of TGF-β1 from M2 macrophages and activated TGF-β signalling (Muraoka et al., 2023). Fusobacterium infection appears to create an environment enriched in TGF-β1 signalling in the endometrium (Chen et al., 2018).

Different theories regarding the pathogenesis of ovarian endometriomas have been proposed including invagination of shed endometrial cells derived from retrograde menstruation into the ovarian cortex and surface epithelial trans differentiation to endometrial-lined ovarian cysts (coelomic metaplasia) (Becker et al., 2022). However, the stimuli responsible for the transformation of coelomic epithelium into endometrial-type glands are still unidentified (Kurita, 2011). Recent research has shown that bacterial infection can induce trans differentiation of epithelial cells during colon tumorigenesis (Kong et al., 2021). Thus, exploring the possibility of Fusobacterium as a trigger for metaplasia warrants further investigation. Further validation of these recent findings is necessary.

While 64% of patients with endometriosis were found to have Fusobacterium in their endometria, suggesting a multifactorial nature of the disease (Muraoka et al., 2023), it is important to note that other bacterial infections may also be involved (Akiyama et al., 2019; Khan et al., 2016; Wessels et al., 2021). The presence of Fusobacterium in both the endometrium and ovarian endometriotic tissues, as well as the observed pathogenic effects of pure cultured F. nucleatum in an in vivo mouse model, support the notion of Fusobacterium having a pathogenic role in endometriosis rather than simply being present in the endometrial environment of reproductive-age women with endometriosis.

However, further research is needed to establish direct evidence linking Fusobacterium presence in the endometrium to endometriosis development after retrograde menstruation.

Based on these observations and assumptions, it is not excluded that antibiotic treatment targeting Fusobacterium in the endometrium, such as Mezlocillin (MZ) or Ciprofloxacin (CP) may offer a potential avenue for improving endometriosis treatment (Muraoka et al., 2023). Clinical studies are necessary to determine the effectiveness of antibiotic treatment against Fusobacterium as a viable therapy for patients with endometriosis. Combining antibiotics with other therapeutic approaches could also be explored in future clinical trials.

## 4. Fertility preservation in endometriosis patients

### 4.1. Why should fertility preservation be considered?

#### 4.1.1. Oocyte quality

Over the last decades extensive research has been carried out investigating a potentially detrimental effect of endometriosis on oocyte quality (Ata et al., 2021).

The presence of endometriosis has been associated with dysregulation of steroidogenesis, leading to an imbalance in oestrogen production (Tummon et al., 1988) as well as an alteration of the intrafollicular milieu (Sanchez et al., 2016). Studies have shown that the dysregulation of intracellular calcium (Ca2+) and increased oxidative stress are likely underlying factors contributing to poor oocyte quality in women with endometriosis (Didziokaite et al., 2023), given that intracellular Ca2+ dysregulation, may lead to failure of the oocyte to maintain metaphase II arrest, aging, and atresia (Da Broi et al., 2018). Women with endometriosis often experience increased oxidative stress, potentially due to compromised antioxidant mechanisms and increased production of ROS in the immediate vicinity of endometriotic implants (Scutiero et al., 2017), and this is believed to be the common pathway for oocyte aging and atresia due to cellular damage (Gao et al., 2023). This is further supported by studies demonstrating elevated nitrate levels in the follicular fluid (FF) of women with endometriosis, indicating rapid generation of peroxynitrite (ONOO−) through the reaction of superoxide (O2·−) and nitric oxide (NO) (Jackson et al., 2005; Scutiero et al., 2017), which may contribute to poor oocyte quality.

On the other hand, endometriosis appears to negatively affect biological markers of oocyte quality. Oocyte morphological characteristics and spindle abnormalities have been reported in patients with endometriosis, as potential contributors to poorer oocyte quality. Goud et al. (2014) revealed that immature oocytes from women with endometriosis exhibited decreased maturation competence and showed signs of cortical granule loss, zona pellucida hardening, and spindle/ chromosome disruption after in vitro maturation (IVM). These abnormalities in unfertilised oocytes may lead to decreased fertilisation and impaired embryo development (Wang et al., 2009).

Nevertheless, despite all the above reports, the actual effect of the presence of endometris on oocyte quality is not completely clear, considering that Juneau et al. (2017) failed to find any difference in embryo aneuploidy rates between women with endometriosis and general population.

#### 4.1.2. Oocyte quantity

Although reports may not be consistent on the actual effect of endometriosis on oocyte quality, accumulating evidence strongly suggests a detrimental effect of endometriosis and endometriosis surgery on ovarian reserve. Two simultaneously published systematic reviews have reported a decrease in anti-Müllerian hormone (AMH) levels after endometrioma excision (Raffi et al., 2012; Somigliana et al., 2012). However, the possibility that the presence of endometriomas per se may impair ovarian reserve has received less attention. Recent meta-analyses demonstrated that patients with ovarian endometriomas have significantly lower AMH levels compared to control patients without endometriomas (Laganà et al., 2020; Muzii et al., 2018; 2014) and retrospective studies have shown that ovarian response after ovarian stimulation for IVF/ICSI in women with endometriomas is significantly lower than in controls (González-Foruria et al., 2020). The above findings may suggest that the mere presence of endometriomas may at least be partly responsible for the reduction in ovarian reserve and response to stimulation (Muzii et al., 2018; 2014), in accordance with histopathological studies showing lower follicular density and increased atresia in ovaries with endometriomas compared to unaffected ovaries (Kitajima et al., 2014; 2011). Although the impact of cyst size and bilaterality on ovarian function has not been specifically addressed, it is reasonable to speculate that larger cysts and bilateral cases may be associated with more severe damage to the ovarian reserve (Busacca et al., 2006; Ferrero et al., 2017).

4.1.3. Clinical practice guidelines and patients counselling for fertility preservation in endometriosis patients

Current clinical practice guidelines do recommend discussion of the risk of reduced ovarian function after endometrioma surgery with patients (Becker et al., 2022), despite available evidence suggesting that the presence of endometriomas alone may lower AMH levels, in addition to the surgical excision (Muzii et al., 2018; 2014). Nonetheless, despite the available guidelines, counselling by reproductive medicine specialists and surgeons is not always consistent. In an online European survey, although 77.6% specialists reported awareness of the existence of endometriosis management guidelines, with 82.2% of them including treatment recommendations for infertile patients, the majority of centres (51.7%) reserved fertility counselling only for severe endometriosis while 15.5% of centres did not offer fertility preservation for endometriosis (Sänger et al., 2023).

### 4.2. When should elective medical fertility preservation for endometriosis be recommended?

Ovarian endometriotic lesions can affect the reproductive capacity of women, and fertility preservation through efficient oocyte vitrification is a viable option for increasing their chances of future motherhood (Tomassetti and D’Hooghe, 2018). This appears to be more relevant in recent years, mainly due to the advances in oocyte cryopreservation and the higher post-thawing survival rates with the introduction of vitrification compared to slow freezing (Rienzi et al., 2017). However, decisions regarding fertility preservation in women with endometriosis are not a simple, since factors such as clinical presentation of the disease, age of the patients and family plans need to be taken into consideration (Yilmaz et al., 2019; Kasapoglu et al., 2018; Uncu et al., 2013).

#### 4.2.1. Endometriosis clinical presentation & fertility preservation

The clinical presentation of endometriosis appears to be of paramount importance for counselling in favour or against fertility preservation. The validity of fertility preservation in different clinical scenarios has been summarised in a systematic review by Somigliana et al. (2015). According to this review, the overall validity of fertility preservation use is dependent on the effect of the clinical presentation on the number and quality of oocytes, the potential detrimental effect of surgery on ovarian reserve and the likelihood that the frozen oocytes will be used. In this context, fertility preservation may be particularly indicated for patients at risk of bilateral ovarian damage, such as women with bilateral endometriomas or patients operated unilaterally with a contralateral recurrence (Somigliana et al., 2015).

#### 4.2.2. Age, family plans & fertility preservation in endometriosis patients

The well-established detrimental effect of age on oocyte quality and quantity appears to play a role in the decision-making process for elective fertility preservation in endometriosis patients.

Advancing female age is associated with lower ovarian reserve (Nelson et al., 2014) and higher aneuploidy rates in the general infertile population (Franasiak et al., 2014). Owing to this strong evidence, oocyte cryopreservation should be offered to women in their 30s if they are not planning motherhood in the following years, and especially if the risk of a negative effect of surgery on ovarian reserve is relevant. Several studies evaluated the association between the number of oocytes retrieved and cumulative live birth rates (CBLR) showing that a higher number of oocytes is needed with advancing maternal age to lead to comparable CLBR. Although evidence concerning the effectiveness of fertility preservation in women with endometriosis is scarce (Cobo et al., 2020), the probability of live birth increases as the number of oocytes used increases in patients with endometriosis, but better outcomes are observed among young women (Cobo et al., 2020). This is also supported by other studies showing that the number of oocytes used per patient is closely related to success in endometriosis patients, whereas young endometriosis patients (≤35 years) who have undergone cystectomy before oocyte retrieval for fertility preservation have worse outcomes than non-operated women in age-matched groups (Cobo et al., 2020). These findings suggest that for young endometriosis patients, it is not advisable to perform surgical excision of endometriotic implants before ovarian stimulation for fertility preservation. On the contrary, in older patients, the impact of surgery on fertility outcomes is less significant, regardless of whether surgery was performed (Cobo et al., 2021; Juneau et al., 2017). In this regard a tailored treatment approach is recommended based on age and disease severity.

Finally, taking into consideration that in severe cases in whom a high number of oocytes for freezing cannot be achieved, the number of cryopreserved oocytes can be increased by repeated oocyte retrieval, to significantly increase success rates (Kim et al., 2020).

Discussing fertility preservation with endometriosis patients before ovarian surgery, appears to be crucial as the results may not support surgery before considering fertility preservation, even at a young age (Streuli et al., 2018). Thus, the high return rate of endometriosis patients in order to use their oocytes following fertility preservation (Cobo et al., 2020) indicates that fertility preservation appears to be relevant for many patients, much more than patients undergoing elective fertility preservation.

## 5. The future new medical therapies for endometriosis

### 5.1. Drug therapies

Hormonal therapies are the primary treatment option for women with endometriosis (Vannuccini et al., 2022). These therapies aim to control the growth of endometrial tissue outside the uterus and alleviate associated pain symptoms. While hormonal therapies cannot cure endometriosis definitively, they are effective in managing the disease and can help prevent or postpone the need for surgery (Allaire et al., 2023).

The main types of hormonal therapies used for endometriosis include progestins (Dunselman et al., 2014), gonadotrophin-releasing hormone agonists (GnRH-a) (Bergqvist et al., 1998; Dlugi et al., 1990; Fedele et al., 1993), and antagonists (Clemenza et al., 2018).

#### 5.1.1. Progestins

Progestins are synthetic compounds that have multiple actions on progesterone receptors. They can reduce pain, suppress endometriosis, and prevent dysmenorrhoea (Reis et al., 2020b).

Dienogest (DNG) is a 19-nortestosterone derivative and is the most recent progestin approved for endometriosis treatment. It has been shown to substantially improve endometriosis-related pain symptoms and is effective in reducing pain associated with different endometriosis phenotypes (Schindler, 2011). DNG also helps reduce the size of ovarian cysts and is effective in controlling pain caused by rectovaginal endometriosis, bladder endometriosis, and DE. It is well-tolerated and does not significantly affect bone mineral density (Liu et al., 2021).

Norethindrone acetate (NETA) is another 19-nortestosterone derivative and is effective in relieving pelvic pain symptoms associated with endometriosis (Ailawadi et al., 2004). Low- dose NETA is commonly used for symptomatic rectovaginal endometriosis and has been shown to decrease pain intensity. Long-term therapy with NETA is safe and well-tolerated, making it a good option for managing endometriosis-related pain (Zito et al., 2014).

Medroxyprogesterone acetate (MPA) is a 17-OH progesterone derivative available in oral and depot formulations. It is as effective as GnRH agonists in reducing endometriosis-related pain (Luciano et al., 1988). Depot MPA is well-tolerated, but long-term use is associated with a risk of bone mineral density loss (Berenson et al., 2008).

#### 5.1.2. GnRH agonists

GnRH agonists (GnRH-a) drugs such as goserelin, leuprolide, nafarelin, buserelin, and triptorelin have been used since the 1990s to treat endometriosis (Surrey, 2023). These drugs initially stimulate the production of luteinising hormone (LH) and follicle-stimulating hormone (FSH), but prolonged exposure leads to downregulation of GnRH receptors, reducing LH and FSH levels and suppressing ovarian oestrogen production (Surrey, 2023). This results in a hypo-oestrogenic state and regression of endometriotic lesions. Treatment with GnRH-a is associated with significant hypo- oestrogenic side effects such as amenorrhoea, vasomotor symptoms, sleep disturbance, urogenital atrophy, and accelerated bone loss (Vannuccini et al., 2022). To mitigate these side effects, add- back therapy is often used, which involves the addition of low-dose COCs, oestrogen or progestins alone, bisphosphonates, tibolone, or raloxifene (Vannuccini et al., 2022). Add-back therapy helps reduce side effects while maintaining pain relief (Surrey, 1999). GnRH-a drugs are effective in relieving pain, but their long-term use should be carefully monitored, especially in adolescents who may not have reached maximum bone density (Divasta et al., 2007).

#### 5.1.3. Other hormonal therapies and alternatives to oral therapies

Several other hormonal therapies are used for endometriosis treatment, although their use may be limited due to side effects or availability.

- Danazol is a derivative of 17α-ethinyl testosterone and has been approved by the FDA since 1971 for endometriosis treatment (Selak et al., 2007). It inhibits pituitary gonadotrophin secretion, suppresses ovarian oestrogen production, modulates immune function, and inhibits endometriotic implant growth. Danazol is effective in reducing endometriosis-related pain, but its use is limited due to androgenic side effects such as seborrhoea, acne, hirsutism, weight gain, liver dysfunction, and osteoporosis (Bhattacharya et al., 2011; Tosti et al., 2017).
- Gestrinone has similar mechanisms of action as danazol and can reduce pain in endometriosis (Brown et al., 2012). However, its use is limited due to androgenic and anti-oestrogenic side effects (Song et al., 2018). Etonogestrel- releasing subdermal implant (ENG-implant) is an effective option for reducing dyspareunia, dysmenorrhoea, and non-menstrual pelvic pain associated with endometriosis (Carvalho et al., 2018; Walch et al., 2009).
- Levonorgestrel intrauterine device (LNG- IUS) has been proven to be effective in relieving pelvic pain symptoms caused by endometriosis and reducing the risk of dysmenorrhoea recurrence after conservative surgery (Viganò et al., 2007). It is a long-acting reversible contraceptive method that releases a low dose of levonorgestrel directly into the uterus (Wattanayingcharoenchai et al., 2021).
- COCs are commonly used off-label for the treatment of endometriosis (ETIC Endometriosis Treatment Italian Club, 2019). They contain synthetic oestrogen and progestin, and their mechanisms of action include reducing menstrual flow, causing endometrial glandular atrophy, and inhibiting ovarian function (Meresman et al., 2002). COCs can help alleviate dyspareunia, dysmenorrhoea, and non-menstrual pain associated with endometriosis (Harada et al., 2017; 2008). However, the evidence for their effectiveness is limited, and about half of the patients may not experience significant improvement in symptoms.

### 5.2. New options of therapies

The need for new options in the treatment of endometriosis arises due to several concerns related to existing medications (Donnez and Dolmans, 2021a).

While oestro-progestins and progestin-only medications provide pain relief and improvement in general condition for two-thirds of symptomatic women, one-third of patients do not respond due to progesterone resistance (Donnez and Dolmans, 2021a). Additionally, there is an increased risk of venous or arterial embolism, and the side effects of oestro-progestins vary depending on the type of progestin used. Furthermore, the reduction in lesion volume is unpredictable and insignificant in most cases, leading to conflicting results.

Selective progesterone receptor modulators (SPRMs) are not a viable option as they induce progesterone modulator-associated endometrial changes (PAECs) in ectopic foci and have limited efficacy (Islam et al., 2020; Murji et al., 2017). There is a lack of randomised controlled trials (RCTs) to evaluate the effect of SPRMs on endometriosis.

The ideal solution would be to lower oestrogen (E2) levels enough to induce amenorrhoea and treat symptoms while maintaining sufficient levels to mitigate severe side effects such as vasomotor menopausal symptoms and bone mineral density (BMD) loss (Donnez and Dolmans, 2021a). Partial suppression of E2 within the range of 30–60 pg/mL could be the optimal compromise between efficacy, tolerance, and safety (Donnez et al., 2017).

Currently, the only option to restore sufficient E2 levels to avoid menopausal symptoms and BMD loss is the combined administration of a GnRH agonist (depot injection) and oestrogens/progestins (add-back therapy) (Chwalisz et al., 2012). GnRH agonists have limitations such as delayed therapeutic impact, excessive E2 suppression, inability to titrate E2 levels, and unpredictable reversibility of treatment (Brown et al., 2010; Dragoman et al., 2016).

Recently, there has been focus on the use of GnRH antagonists, which competitively block the GnRH receptor, suppress production of FSH and LH, and inhibit secretion of ovarian steroid hormones without inducing a flare-up effect (Al-Inany et al., 2016; Donnez and Dolmans, 2021a). GnRH antagonists offer dose-dependent oestrogen suppression, rapid reversibility, and the potential for individual tailoring of treatment (Kumar and Sharma, 2014).

- Elagolix is a GnRH antagonist that has been approved by the FDA. It effectively reduces dysmenorrhoea, non-menstrual pelvic pain, and dyspareunia in women with endometriosis (Leyland et al., 2021; Taylor et al., 2017). However, it causes dose-dependent decreases in bone mineral density (BMD), and long-term use may require hormone add-back therapy (Donnez and Dolmans, 2021a).
- Linzagolix is another GnRH antagonist that has shown efficacy in reducing endometriosis- associated pain (Donnez et al., 2023). It provides partial suppression of E2 levels and has a significant impact on dyspareunia and certain aspects of quality of life. However, higher doses may lead to more hypo-oestrogenic symptoms and BMD loss, necessitating add-back therapy for longer-term use (Donnez and Dolmans, 2021a).
- Relugolix is an oral GnRH antagonist that has demonstrated efficacy in reducing pelvic pain associated with endometriosis (Giudice et al., 2022). It is well-tolerated and maintains bone mineral density over 24 weeks of treatment (Donnez and Dolmans, 2021a).
- Desogestrel is an effective, safe, and low- cost therapy for endometriosis-related pain. It has been shown to significantly decrease pain symptoms and improve quality of life (Morotti et al., 2014).
  Studies have shown that oestrogen receptor alpha (ERα) action is reduced, while oestrogen receptor beta (ERβ) activity is upregulated in endometriotic implants, leading to the loss of progesterone receptor B (PR-B) and high levels of oestrogen (E2) (Trukhacheva et al., 2009). Progesterone resistance in adult women is believed to be influenced by factors such as inflammation and oxidative stress (Fedotcheva et al., 2022).

New treatment options are needed due to concerns about the effectiveness of current drugs. Oestro- progestins and progestin-only medications may not be effective for all patients due to progesterone resistance (Donnez and Dolmans, 2021a). Selective progesterone receptor modulators (SPRMs) are not suitable options, and GnRH agonists have limitations such as delayed therapeutic impact and unpredictable reversibility (Donnez and Dolmans, 2021b). Achieving partial E2 suppression while maintaining adequate levels for symptom relief and minimising side effects is desirable.

#### 5.2.1. GnRH Antagonists with or without Add-Back Therapy

The effectiveness of oestro-progestins and progestins in treating endometriosis varies among women (Donnez and Dolmans, 2021b). Casper (2017) suggests that progestin-only pills are a better first-line treatment than oestro-progestins. However, Vercellini et al. (2018a; 2018b) argue that progestin-only therapy should be reserved for women who have contraindications or intolerance to oestro-progestins. Despite being included in various guidelines, the use of oral contraceptive pills (OCPs) containing oestro-progestins is considered off-label (Dayal and Barnhart, 2001).

GnRH antagonists, such as elagolix, suppress gonadotropin hormone production by competing with endogenous GnRH for its pituitary receptors (Clemenza et al., 2018). Elagolix has been approved for the management of moderate to severe pain associated with endometriosis (Agarwal et al., 2021). It provides pain relief without causing severe hypo-oestrogenism. However, it can still have side effects such as hot flushes, decreased bone mineral density, and increased serum lipid levels (Donnez et al., 2020). Other GnRH antagonists like relugolix and linzagolix are also being investigated and have shown efficacy in alleviating endometriosis- associated pain (Rzewuska et al., 2023).

Studies have shown that about 33% of patients treated with oestro-progestins and/or progestins do not respond to therapy (Casper, 2017; Vercellini, 2018; Vercellini et al., 2016). The efficacy of OCPs in treating endometriosis-related pain is limited, and there is no significant beneficial effect on non- menstrual pelvic pain or dyspareunia (Brown et al., 2018). Additionally, there is a lack of data on the efficacy of OCPs based on lesion phenotype.

Different progestins, including norethisterone acetate (NETA), dienogest, desogestrel, cyproterone acetate, depot medroxyprogesterone acetate (DMPA), and the levonorgestrel-releasing intrauterine system (LNG-IUS), have been used in the management of endometriosis. Studies suggest that all available progestins are equally effective in controlling pain symptoms in about two-thirds of women with endometriosis, and there is no evidence to suggest the superiority of one progestin over another (Barbara et al., 2021). NETA is recommended as a first-line treatment due to its favourable cost-effectiveness profile (Barbara et al., 2021). However, a substantial proportion of patients (~30%) may be dissatisfied with progestin therapy (Donnez and Dolmans, 2021b).

Thus, a combined symptom-oriented and phenotype-adapted approach is necessary in the management of endometriosis. Treatment options should be tailored based on the main symptoms and different phenotypes of endometriosis. First-line therapy with OCPs or progestins can be considered, but poor response and drug intolerance may require the use of GnRH antagonists. Surgical intervention may be necessary for specific cases, such as endometriomas and deep nodular endometriosis. Further research is needed to evaluate the role of GnRH antagonists in different endometriosis phenotypes and the long-term benefits of these treatments.

#### 5.2.2. Selective progesterone receptor modulators (SPRMs)

The functions of the endometrium are strongly influenced by two key steroid hormones, E2 and P4 (Islam et al., 2020). These hormones play a vital role in regulating the expression of numerous genes throughout the menstrual cycle (Kao et al., 2002). While E2 signalling is considered a major factor in the development and growth of endometriosis (Bulun, 2009; Yilmaz and Bulun, 2019), P4 has an opposing effect (Li et al., 2016). Progesterone resistance is believed to contribute to the development of endometriosis (McKinnon et al., 2018). The balance between E2 and P4 levels can be altered by the local expression of enzymes, which in turn can affect the activation or inhibition of progesterone receptors (PR) in the disease state. One important mediator in this process is the HSD3B enzyme, which converts dehydroepiandrosterone into androstenedione, a precursor of oestrogen production. Higher expression and activity of HSD3B2 mRNA has been observed in endometriotic tissue compared to normal endometrium (Huhtinen et al., 2014), indicating elevated E2 levels in endometriosis. Conversely, lower expression of CYP11A1, which is involved in P4 synthesis, has been observed in endometriotic lesions (Huhtinen et al., 2014), suggesting decreased P4 production in these tissues. Huhtinen et al. (2012) reported significantly lower expression of HSD17B2 and significantly higher expression of HSD17B6 and CYP19A1 in endometriotic lesions compared to endometrial tissue.

In endometriotic lesions, the expression of PR-A is reduced, and PR-B is absent compared to normal endometrium (Attia et al., 2000). Additionally, several P4-regulated genes, such as glycodelin, N-acetylglucosamine-6-O-sulfotransferase, and 17β hydroxysteroid dehydrogenase 2 (17βHSD2), have been found to be decreased in the eutopic endometrium of individuals with endometriosis (Burney et al., 2007). Within the endometrium, P4 stimulates the expression of 17βHSD2, an enzyme that converts biologically potent oestradiol to the less estrogenic oestrone (Yang et al., 2001). P4 can induce the production of retinoic acid by endometrial stromal cells, which, in turn, promotes the expression of 17βHSD2 in endometrial epithelial cells through paracrine signalling (Cheng et al., 2008). However, endometriotic stromal cells do not respond to P4, leading to a lack of retinoic acid production in these cells (Cheng et al., 2007). This deficiency in retinoic acid results in reduced epithelial 17βHSD2 expression and the failure to deactivate oestradiol in endometriotic tissues (Cheng et al., 2007). The inability of endometriotic tissues to upregulate 17βHSD2 in response to P4 may be attributed to decreased expression of PR-B in stromal cells. Indeed, the loss of PR expression or disruption of the PR- mediated signalling pathway is often associated with excessive E2 activity in the endometrium and the development of gynaecological conditions, including endometriosis (Tangen et al., 2014). A recent study demonstrated that treating female mice with P4 before inducing endometriosis inhibited the development and growth of ectopic lesions, primarily by reducing cell proliferation, inflammation, and angiogenesis (Li et al., 2016). Consequently, the antiendometriotic properties of P4 have led to the use of progestins as hormonal therapies for the clinical treatment of endometriosis (Vercellini et al., 2014). Unfortunately, the therapeutic potential of P4 in managing endometriotic patients remains challenging due to the proliferative effects of P4 on endometrial stromal cells (Vallejo et al., 2014), which constitute a major cellular component in ectopic lesions. Clinical and translational studies indicate that endometriosis is a complex condition, and while some ectopic endometrial lesions respond to P4 therapy, others may be resistant (Flores et al., 2018). Further research is needed to understand the basis of P4 resistance and identify the underlying factors that downregulate PR signalling pathways in these diseased tissues.

#### 5.2.3 Neurokinin receptor antagonists

There is a need for new approaches that can effectively and dose-dependently reduce oestradiol levels to target concentrations (Rosner et al., 2013). Recent research has established that the secretion of gonadotropin-releasing hormone (GnRH) is regulated by specific neurons in the hypothalamus known as KNDy neurons, which express kisspeptin, neurokinin B (NKB), and dynorphin (Uenoyama et al., 2021). NKB stimulates the secretion of GnRH through the neurokinin 3 receptor (NK3R), while substance P (SP) acts on the NK1 receptor (NK1R) to stimulate GnRH activity (Gaskins et al., 2013). Blocking these receptors has been shown to reduce GnRH pulsatility and lower gonadotropin and oestradiol levels in women (Tsutsumi and Webster, 2009). Elinzanetant, a dual NK1R and NK3R antagonist, has the potential to reduce GnRH pulsatility by blocking the effects of NKB and SP on the reproductive axis, leading to decreased LH and subsequently lower oestradiol levels (Pawsey et al., 2021).

In a clinical study involving healthy premenopausal women, oral administration of elinzanetant at different doses over a full menstrual cycle resulted in a dose-dependent reduction in serum LH, oestradiol, and progesterone levels, particularly during the luteal phase (Pawsey et al., 2021). Additionally, there was a dose-related increase in serum FSH, although statistical significance was not achieved (Lawrenz et al., 2021). The proportion of women with a progesterone level consistent with ovulation was significantly reduced with elinzanetant treatment in a dose-dependent manner, suggesting an increased rate of anovulation (Pawsey et al., 2021). The length of the menstrual cycle was also significantly increased with elinzanetant. These effects align with the expected outcomes of NK1R and NK3R antagonism on reproductive hormone secretion (Pawsey et al., 2021).

The findings of this study indicate that elinzanetant has the potential to effectively lower oestradiol levels throughout the menstrual cycle (Pawsey et al., 2021). Maintaining oestradiol within a therapeutic range is crucial for conditions such as endometriosis (EM), as it can suppress the growth of these hormonally responsive tissues while minimising adverse symptoms and long-term effects on bone health and cardiovascular risk (Barbieri, 1992). Elinzanetant may offer a novel therapeutic approach to achieve the desired reduction in hormonal drive to the endometrium or myometrium without compromising bone health. Further studies are needed to assess the long-term effects and optimal dosing of elinzanetant on oestrogen levels. While this study has several strengths, including a randomised, single-blinded, placebo-controlled design, standardised assessments, and good compliance, it also has limitations such as a small sample size, limited frequency of hormone sampling, and short duration (Pawsey et al., 2021). Future studies should include larger and longer investigations to evaluate the effects of elinzanetant on hormone pulsatility, as well as imaging to assess endometrial thickening and follicle growth. The potential risk of unopposed oestrogen exposure and endometrial hyperplasia should also be further evaluated. Overall, these findings provide valuable insights into the use of elinzanetant as a therapy for hormone-driven disorders and support the need for further research in this area.

#### 5.2.4. Stem Cells therapies

Stem cell therapy holds promise as an innovative approach for the treatment of endometriosis (Liu et al., 2023). Stem cells can be derived from various sources, including adipose tissue, umbilical cords, embryos, bones, gums, and menstrual blood (Ding et al., 2011). Based on their differentiation potential, stem cells can be categorised into different types, such as totipotent, pluripotent, multipotent, oligopotent, and unipotent (Zakrzewski et al., 2019). One of the main types of stem cells studied for endometriosis therapy is mesenchymal stem cells (MSCs) (Rungsiwiwut et al., 2020). MSCs have demonstrated multiple physiological functions, including the ability to differentiate into various cell types, promote tissue repair, and modulate inflammation and the immune response (Wang et al., 2018). These cells possess self-renewal properties and can home in damaged tissues, where they aid in tissue regeneration and replace damaged cells. Additionally, MSCs secrete bioactive factors such as chemokines, growth factors, and cytokines, which contribute to the regeneration process (Han et al., 2022). MSCs are considered a promising approach for regenerative medicine due to their easy isolation from different tissues, in vitro amplification capabilities, and low immunogenicity, enabling their use as allografts (Han et al., 2019).

Endometrial stem cells (EnSCs) have also been implicated in the pathogenesis of endometriosis (Sasson and Taylor, 2008). EnSCs residing in the basal layer of the endometrium exhibit unique characteristics in endometriosis patients (Liu et al., 2020). These cells demonstrate prolonged mitosis, enhanced migration, and increased angiogenesis potential compared to EnSCs from unaffected individuals (Liu et al., 2020). The study of EnSCs isolated from endometriosis lesions provides valuable insights into the development and progression of endometriosis, potentially leading to the identification of therapeutic targets and biomarkers (Brichant et al., 2021).

Induced pluripotent stem cells (iPSCs) have emerged as an alternative to embryonic stem cells for research and therapeutic purposes (Wu and Hochedlinger, 2011). iPSCs can be generated by reprogramming somatic cells using specific factors (Patel and Yang, 2010). These cells retain the properties of self-renewal and pluripotency and can differentiate into various cell types. iPSCs offer the advantage of being derived from patient- specific cells, reducing ethical concerns associated with embryonic destruction (Patel and Yang, 2010). They can serve as models to study the molecular mechanisms of endometriosis development in specific cell types and facilitate drug screening. iPSCs have the potential to differentiate into endometrial mesenchymal fibroblasts (EMSF), which play a crucial role in the interaction between stromal and epithelial cells in the endometrium (Miyazaki et al., 2018). EMSF replacement therapy using iPSC-derived EMSF holds promise for restoring progesterone responsiveness and treating endometrial diseases such as endometriosis and uterine factor infertility (Liu et al., 2023).

While stem cell therapy shows potential, there are several clinical challenges that need to be addressed. Ensuring the safety and efficacy of stem cell transplantation is of utmost importance. Further validation by clinical trials is necessary. Success rates of spontaneous conception vary among patients, and factors such as the duration of amenorrhoea and ovarian condition may influence therapeutic outcomes. Immune responses, immune rejection, clotting, and tumorigenesis are potential side effects associated with stem cell therapy (Herberts et al., 2011). Long-term observations and identification of risk factors and risk populations are crucial for a comprehensive understanding of the therapy’s safety profile.

In conclusion, stem cell therapy, particularly using MSCs, EnSCs, and iPSC-derived cells, holds promise for the treatment of endometriosis. These cells offer regenerative, anti-inflammatory, and immunomodulatory properties that can target endometriotic lesions and restore normal tissue function. However, further research, larger-scale clinical trials, and long-term safety evaluations are necessary to establish the effectiveness, safety, and optimal protocols for stem cell therapy in endometriosis patients.

### 5.3. Immunotherapies

Immunotherapy has emerged as a potential strategy for the treatment of endometriosis (Li et al., 2023). Accumulating evidence suggests that immune factors play a significant role in the pathogenesis of this disease, and targeting the immune system may offer promising therapeutic opportunities (Chen et al., 2023). Various immunocompetent cells have been implicated in the development of endometriosis, including neutrophils, macrophages, NK cells, T cells, mast cells, dendritic cells, and others (Vallvé-Juanico and Giudice, 2022).

Neutrophils, a type of white blood cell, are elevated in the abdominal cavity of endometriosis patients, particularly during the early stages of the disease (Milewski et al., 2011). Their aggregation is believed to be driven by increased concentrations of chemokines such as IL-8, ENA-78, and HNP1- 3. Neutrophil aggregation is associated with acute inflammatory reactions and may contribute to pelvic pain in affected patients (Milewski et al., 2011).

Macrophages, another type of immune cell, are also increased in the peritoneal fluid and endometrial tissue of endometriosis patients (Bacci et al., 2009). They play a role in inducing inflammatory reactions, promoting endometrial cell proliferation, facilitating angiogenesis in endometriosis lesions, and impairing phagocytosis (Hogg et al., 2020). Different types of macrophages, such as MI and MII phenotypes, have been implicated in the development of endometriosis (Hogg et al., 2020).

Several immunotherapy strategies have been explored for endometriosis treatment. These include immune cell inhibitors or stabilisers to restore immune balance, immune cytokine modulators to regulate inflammatory factors, complement system inhibitors to block inflammatory signal cascades, and other immunomodulators such as mesenchymal stem cells and vitamin D. Each of these approaches targets specific aspects of the immune system to alleviate endometriosis symptoms and inhibit disease progression.

## 6. Endometriosis: hormone substitution or not after radical treatment...this is the question!

### 6.1. Endometriosis after menopause

The prevailing notion that endometriosis only affects women of reproductive age has been challenged by evidence showing its occurrence in various age groups (Haas et al., 2012). While the majority of cases are still reported in reproductive-age women, there have been documented cases of endometriosis in postmenopausal women (Secosan et al., 2020). Notably, a retrospective epidemiological study found that out of 42,079 women admitted for surgical treatment of endometriosis with histological proof of the disease, 2.55% were in the postmenopausal group (Haas et al., 2012).

Numerous studies have described the recurrence of endometriosis lesions after menopause in women with a previous diagnosis of the condition during the premenopausal period. Additionally, cases of de novo appearance of endometriosis in postmenopausal women with no prior history of the disease have also been reported (Secosan et al., 2020). Although the presence of previous undiagnosed endometriosis is still a possibility, this challenges the notion that endometriosis is solely a disease of reproductive age and highlights the need for further understanding of its prevalence and pathophysiology in postmenopausal women.

The prevalence of postmenopausal endometriosis is estimated to be approximately 2-5% and is commonly associated with hormone replacement therapy (HRT) (Haas et al., 2012; Smolarz et al., 2021). However, there have been rare cases of postmenopausal endometriosis reported in women who were not administered HRT or tamoxifen treatment.

The pathophysiology of postmenopausal endometriosis is still not well understood. Excess oestrogen, either from peripheral production in adipose tissue and skin (Bulun, 2009) or from external sources like phytoestrogens and HRT, is thought to play a role in promoting endometriosis (Jeon et al., 2013). However, the exact mechanisms and triggers for the development of the disease in postmenopausal women remain unclear.

The clinical presentation of endometriosis in menopausal patients is often nonspecific, with symptoms such as pelvic pain, ovarian cysts, or intestinal symptoms (Cope et al., 2020). Differentiating between endometriosis and other conditions, including malignancy, can be challenging, and a thorough evaluation is necessary to rule out any potential neoplastic process.

The diagnosis of endometriosis in postmenopausal women is complicated by the lack of non-invasive diagnostic tools (Secosan et al., 2020). Laparoscopy and biopsy for histological confirmation are still considered the gold standard for diagnosis, regardless of age. Imaging techniques such as MRI and ultrasound (Alborzi et al., 2018; Vimercati et al., 2012) can be helpful but are more challenging to interpret in older patients due to the higher susceptibility to neoplastic lesions and the varied appearance of endometriosis.

Additionally, distinguishing between endometriomas and malignant ovarian tumours in postmenopausal women remains challenging, and novel tests and biomarkers for endometriosis have not yet demonstrated reliable diagnostic utility (Han et al., 2018; Dorien et al., 2019).

Extrapelvic endometriosis, while rare, has also been reported in postmenopausal women. It most commonly affects the gastrointestinal and urinary tracts, with locations such as the sigmoid colon, bladder, and ureter being frequently involved (Popoutchi et al., 2008; Secosan et al., 2020). Confirmation of the diagnosis usually requires surgical exploration.

Surgical intervention through laparoscopy is thus often necessary for both diagnosis and treatment of endometriosis. Complete resection of visible lesions is recommended, particularly in postmenopausal women, to reduce the risk of recurrence and potential malignant transformation of endometriotic lesions (Ozyurek et al., 2018). Medical therapy can be considered for pain management provided that prior surgical investigations were reassuring in terms of malignant transformation or risk for, or when surgery is contraindicated (Oxholm et al., 2007; Ozyurek et al., 2018; Streuli et al., 2017).

While tamoxifen, a hormonal therapy used in postmenopausal women with breast cancer, has been associated with endometriosis development, the exact mechanisms are not fully understood (Hajjar et al., 1993) and the risk of malignant transformation of endometriosis lesions in postmenopausal women who have received tamoxifen also remains debatable (Inceboz, 2015). Hence, further studies are needed to clarify this association.

Management of climacteric symptoms in postmenopausal women with a history of endometriosis remains controversial due to concerns about disease reactivation, recurrence of symptoms during or after HRT and risk of malignant transformation under hormone exposure. The choice of the most suitable hormone therapy should be carefully evaluated as risks may be regimen dependent. As discussed below, current evidence is poor regarding optimal and safe hormone administration, which thus calls for larger studies (Streuli et al., 2017).

### 6.2. HRT in patients with a history of endometriosis

A Cochrane review in 2009 evaluating this matter identified only two randomised clinical trials, suggesting that HRT may increase the risk of symptomatic recurrence after surgically induced menopause (Al Kadri et al., 2009). Notably among the case reports, a consistent finding is the use of oestrogen-only hormone replacement therapy (HRT) in women with endometriosis recurrence or malignancy after menopause whereas there are fewer reports where combined hormone therapy was administered (Gemmell et al., 2017). Based on this observation and the strong association between unopposed oestrogens and endometrial cancer (Sjögren et al., 2016), current recommendations lean towards continuous combined HRT instead of unopposed oestrogens for women with a history of endometriosis, although the evidence supporting this is limited (Becker et al., 2022). In addition, some randomised studies suggested that combined HRT preparations might be more suitable for women with endometriosis using HRT (Rattanachaiyanont et al., 2003). However, large, randomised trials or observational studies with adequate statistical power are necessary to provide clearer answers and allow a better evaluation of the risk-benefit balance taking into account the increased risk of breast cancer associated with combined HRT, which has been attributed to progestins (Chlebowski et al., 2013).

Tibolone therapy has also been linked to endometriosis recurrence (Sundar et al., 2007). Fedele et al. (1999) concluded that tibolone, which has oestrogenic effects on climacteric symptoms and bone but a tissue-specific progestogenic effect on endometrium, might be a safer alternative to traditional HRT in patients with residual endometriotic disease, but no statistically significant difference was observed between the groups, although this randomized control trial only included 21 patients.

Importantly, a case report highlights the significance of inquiring about patients’ use of nutritional supplements or alternative medications. In this regard, the prolonged use of a highly concentrated isoflavone supplement for five years was associated with the recurrence of endometriosis and a rare form of malignant mullerian carcinosarcoma in the ureter (Noel et al., 2006). This report raises concerns about the use of phytoestrogens in postmenopausal women with a history of endometriosis, despite some clinical and animal studies suggesting a reduced risk of endometriosis with dietary isoflavones (Bartiromo et al., 2021). Due to the widespread use of supplements, further investigation is necessary to explore the relationship between phytoestrogens and endometriosis.

Regarding the timing of HRT initiation and duration, there is a lack of data on the optimal time to start HRT after surgical menopause. A retrospective study comparing immediate initiation (within 6 weeks of surgery) with delayed initiation (≥6 weeks after surgery) found no difference in the crude incidence of recurrence (Hickman et al., 1998). However, increased recurrence was observed in women who delayed starting HRT after adjusting for confounding factors. The observational nature of the study introduces the likelihood of bias, as deferring HRT initiation would likely be recommended to women deemed at higher risk of recurrent symptoms. Randomised trials are clearly needed to avoid bias and provide a robust answer to this question. Unfortunately, no studies were found that investigated the optimal duration of HRT treatment for women with a history of endometriosis.

#### 6.2.1. Recurrence rate of endometriosis: studies on HRT including a control group

When making decisions about hormone replacement therapy (HRT) for postmenopausal symptomatic women with a history of endometriosis, various factors should be considered, including the presence of residual disease and the severity of symptoms (Zanello et al., 2019). The main concern in this context is the possibility that exogenous oestrogen stimulation could reactivate endometriotic lesions, as well as the risk of malignant transformation of the lesions (see 6.2.2) (Gemmell et al., 2017).

Limited research has been conducted on HRT in women with a history of endometriosis, primarily focusing on those who underwent hysterectomy and bilateral salpingo-oophorectomy (BSO) for symptomatic endometriosis. Gemmell et al. (Gemmell et al., 2017) in their systematic review found only one randomized clinical trial and two cohort studies assessing the risk of endometriosis recurrence and comparing postmenopausal women using HRT versus those not using therapy.

In the randomised clinical trial conducted by Matorras et al. (2002), which included 172 women with a history of endometriosis who underwent BSO, participants were assigned to receive combined HRT or no treatment. Recurrences of endometriosis were observed exclusively in the HRT group (3.5%), but the difference in recurrence rates between the two groups was not statistically significant. The authors suggested that the presence of residual endometrial tissue might be a risk factor for recurrence.

Acién et al. (2013) described 19 patients who underwent hysterectomy and BSO for symptomatic endometriosis. Among them, 11 received HRT (1-2 years of combined HRT, followed by low- dose oestrogen-only HRT or tibolone), while eight did not receive hormonal therapy. None of the patients in either group experienced endometriosis recurrence. In another retrospective cohort study, 107 women who underwent hysterectomy and BSO for endometriosis were included. Among them, 90 received HRT (including various regimens with unopposed oestrogens or combined HRT), while 17 were not treated (Rattanachaiyanont et al., 2003). Recurrence was observed in four women receiving unopposed oestrogen therapy.

#### 6.2.2. Malignant transformation of endometriotic lesions: impact of HRT and risk factors for endometriosis-associated carcinoma

Endometriosis, a benign condition, may carry a risk of malignant transformation, although the incidence is low. Studies have reported that approximately 1% of ovarian endometriosis cases can progress to cancer (Melin et al., 2006; Stern et al., 2001). However, a prospective study with a mean follow-up of 13 years involving around 6500 women with ovarian endometrioma showed a standardised incidence ratio of 8.95 for malignant transformation (Kobayashi, 2009). While malignant transformation is likely to be infrequent in the context of infertility care, the perimenopausal period, typically between 45-49 years of age, has been identified as a critical time when the risk of malignant transformation increases for endometriotic ovarian cysts (Kobayashi, 2009; Kobayashi et al., 2008; Modesitt et al., 2002). Another important concern in this population is the role that HRT may play in the development of endometriosis associated neoplastic lesions. Hence, close attention should be paid to endometriosis in peri and post-menopausal women, especially when it involves ovarian cysts.

Malignant transformation of endometriotic lesions in postmenopausal women is rare, and its prevalence is not well-defined as available data come from case reports and case series.

A systematic review by Gemmell et al. (2017) identified 25 cases of postmenopausal malignant transformation in women using hormone replacement therapy (HRT) with a previous history of endometriosis. The majority of patients (88%) had undergone surgical menopause, and 76% of them used oestrogen-only HRT for an average duration of approximately seven years (ranging from 3 to 20 years). Common clinical presentations included abdominal/pelvic pain, vaginal bleeding, and palpable masses. Endometrioid adenocarcinoma was the most frequent histological subtype, and surgical treatment followed by adjuvant therapy was the primary approach. The follow-up outcomes were generally favourable, with a mortality rate of 12% over an average observation period of 19.4 months.

Another report by Tan and Almaria (2018) reported 62 cases of malignant transformation of endometriosis in menopause. The mean age at diagnosis was 58.2 years, and approximately half of the women had a history of HRT use, with 71% using unopposed oestrogen therapy. The average duration of HRT use was ten years, and the predominant histological subtype was endometrioid adenocarcinoma (67.7%).

A recent systematic review (Giannella et al., 2021) analysing 90 patients whose full case description of malignant transformation of endometriosis- related lesions was available, provides extensive and updated data on this topic. Some recurrent clinical conditions associated with malignant transformation include a history of endometriosis/ adenomyosis (60% of cases), definitive surgical treatment before menopause (such as hysterectomy with salpingo-oophorectomy in 57% of cases), and the use of oestrogen-only HRT (73% of cases). The available follow-up data show favourable survival rates of approximately 80% over an average observation period of 12 months (ranging from 6.75 to 25 months) (Giannella et al., 2021).

It has been speculated that women with postmenopausal malignant transformation of endometriosis presented with the most severe endometriosis that eventually led to definitive surgical intervention before menopause. This may also explain the high prevalence of oestrogen- only HRT user for extended periods in this group (Giannella et al., 2021). Interestingly, the results of this study also indicate that in 36% of the patients with malignant transformation, no previous diagnosis of endometriosis had been made, suggesting that the condition may have been unrecognised preoperatively or intraoperatively. This is in line with previous studies that have shown that rectovaginal endometriosis is often overlooked during initial surgeries (Griffiths et al., 2007) and points to the need of expert surgeons to avoid residual lesions after surgery.

Another significant observation is the high percentage (58.4%) of previous bilateral salpingo- oophorectomy among women with subsequent malignant transformation (Giannella et al., 2021). This underscores that “risk-reducing surgical treatment” in women with previous endometriosis approaching menopause may not be cost-effective (Vercellini et al., 2018c). Instead, emphasis should be placed on removing all detected endometriotic lesions during surgery to reduce the risk of recurrence and malignant transformation and obtain a histological diagnosis (Becker et al., 2022).

The exact mechanism underlying malignant transformation remains unclear. While it is possible that microscopic endometriotic foci may be overlooked and left behind during surgical treatment, surgery itself could create an inflammatory stimulus that promotes the implantation or activation of undetected endometriotic foci. In cases where surgery is not performed, asymptomatic endometriosis lesions may persist for extended periods and receive autocrine, paracrine, and exogenous stimuli along with cancer-predisposing gene mutations (Marie- Scemama et al., 2019; Vercellini et al., 2018c).

In summary, based on scant literature, current recommendations favour continuous combination formulations or Tibolone in women with a history of endometriosis (Becker et al., 2022). Besides treating severe climacteric symptoms, the prescription of HRT should carefully consider the risks of bone and cardiovascular diseases alongside the potential for recurrence and malignant transformation of endometriotic lesions (Gemmell et al., 2017). However, solid evidence based on extensive studies is lacking to provide definitive guidelines.

While malignant transformation of endometriosis in postmenopausal women is rare, it necessitates vigilant management and surveillance, particularly in those with a history of severe endometriosis and those using HRT. Further research, including randomised controlled trials and comprehensive observational studies, is required to gain a better understanding of the risks, outcomes, and optimal treatment approaches for postmenopausal women with endometriosis.

## Conclusion

Six main topics on endometriosis disease and management explored in this paper shed light on the future directions of endometriosis classification, diagnosis, and therapeutical management. The first question addressed the possibility of preventing endometriosis in the future by identifying risk factors. Furthermore, the clinical presentation of endometriosis is varied, and the correlation between symptoms severity and disease extent remains unclear. While there is currently no universally accepted optimal classification system for endometriosis, the several attempts striving towards its optimisation, each with its own advantages and limitations, should be investigated in future studies. Thus, the ideal classification should be able to reconcile disease status based on the various diagnostic tools, and prognosis considering patients expectations i.e. treating either infertility or other pain-related symptoms, to guide effective patient tailored management. Besides current treatment modalities, potential novel medical therapies are required that target underlying mechanisms, provide effective symptom relief, and minimise side effects in endometriotic patients.
